# Neonatal Outcomes in Pregnant Women Infected with COVID-19 in Babol, North of Iran: A Retrospective Study with Short-Term Follow-Up

**DOI:** 10.1155/2021/9952701

**Published:** 2021-06-02

**Authors:** Zahra Akbarian-Rad, Mohsen Haghshenas Mojaveri, Zinatossadat Bouzari, Farzin Sadeghi, Yousef Yahyapour, Mojgan Naeimi Rad, Somayeh Alizadeh, Soheil Ebrahimpour, Mahdi Sepidarkish, Mostafa Javanian

**Affiliations:** ^1^Non-Communicable Pediatric Disease Research Center, Health Research Institute, Babol University of Medical Sciences, Babol, Iran; ^2^Department of Obstetrics & Gynecology, School of Medicine, Babol University of Medical Sciences, Babol, Iran; ^3^Infertility and Reproductive Health Research Center, Health Research Institute, Babol University of Medical Sciences, Babol, Iran; ^4^Cellular and Molecular Biology Research Center, Health Research Institute, Babol University of Medical Sciences, Babol, Iran; ^5^Infectious Diseases and Tropical Medicine Research Center, Health Research Institute, Babol University of Medical Sciences, Babol, Iran; ^6^Clinical Research Development Center, Ayatollah Rouhani Hospital, Babol University of Medical Sciences, Babol, Iran; ^7^Department of Biostatistics and Epidemiology, School of Public Health, Babol University of Medical Sciences, Babol, Iran

## Abstract

During the coronavirus disease 2019 (COVID-19) pandemic, the number of pregnant women and neonates suffering from COVID-19 increased. However, there is a lack of evidence on clinical characteristics and neonatal outcomes in pregnant women with COVID-19. We evaluated short-term outcomes (4 weeks postdischarge) and symptoms in neonates born to mothers infected with COVID-19. In this retrospective cohort study, we included all neonates born to pregnant women with COVID-19 admitted to Ayatollah Rohani Hospital, Babol, Iran, from February 10 to May 20, 2020. Clinical features, treatments, and neonatal outcomes were measured. Eight neonates were included in the current study. The mean gestational age and birth weight of newborns were 37 ± 3.19 weeks (30^₊6^-40) and 3077.50 ± 697.64 gr (1720-3900), respectively. Apgar score of the first and fifth minutes in all neonates was ≥8 and ≥9 out of 10, respectively. The most clinical presentations in symptomatic neonates were respiratory distress, tachypnea, vomiting, and feeding intolerance. This manifestation and high levels of serum C-reactive protein (CRP) in three infants are common in neonatal sepsis. The blood culture in all of them was negative. They have been successfully treated with our standard treatment. Our pregnant women showed a pattern of clinical characteristics and laboratory results similar to those described for nonpregnant COVID-19 infection. This study found no evidence of intrauterine or peripartum transmission of COVID-19 from mother to her child. Furthermore, the long-term outcomes of neonates need more study.

## 1. Introduction

Coronavirus disease 2019 (COVID-19) pandemic is known to have originated from Wuhan, China, in December 2019 [1–3]. This unique viral infection, officially known as severe acute respiratory syndrome coronavirus 2 (SARS-CoV-2), commonly causes severe human symptoms. By August 30, 2020, there have been 24,854,140 confirmed cases of COVID-19, including 838,924 deaths worldwide [4]. The pandemic has spread to 188 countries around the world. In addition to the rapid spread of infection, the number of pregnant women and neonates with COVID-19 has also increased [5–7]. In a study identified in Turkey, maternal infection with COVID-19 increases the rate of pregnancy complications (miscarriage, preterm delivery) [8]. Evidence suggests that elevated levels of inflammatory cytokines during infection with COVID-19 and changes in the balance of inflammatory and anti-inflammatory cytokines in pregnant women can cause pregnancy complications [9]. Though information is not enough on clinical features and neonatal outcomes in pregnant women with COVID-19, we assessed short-term outcomes (4 weeks after discharge) and symptoms among neonates born to mothers infected with COVID-19 at the end of the first regional peak of the outbreak.

## 2. Materials and Methods

### 2.1. Study Design, Ethical Considerations, and Participants

In the current retrospective cohort study, all pregnant women suspected of contracting COVID-19 were admitted to Ayatollah Rohani Hospital, Babol, Iran, for delivery between February 10 and May 20, 2020. This study protocol was approved by the Ethics Committee of Babol University of Medical Sciences, Babol, Iran (Code: IR.MUBABOL.REC.1399.092). Also, written informed consent was obtained from the patients. All women with COVID-19 enrolled in the recent research were diagnosed and managed following WHO interim guidance for 2019 novel coronavirus [10]. In other words, all women with laboratory-confirmed (positive in nasopharyngeal/throat swab specimens by reverse transcription-polymerase chain reaction (RT-PCR)) COVID-19 infection or suggestive findings on high-resolution computed tomography (HRCT) of the chest were included. At the same time, suspected patients with similar symptoms were excluded from the study. According to the instructions of the time, a cesarean section was recommended unless vaginal delivery progressed rapidly.

Due to insufficient evidence, a cesarean section was recommended to minimize the mother suspected of having COVID-19 in labor and reduce the risk of infection acquisition. Newborns were also monitored following national guidelines. Nasopharyngeal swabs for RT-PCR were obtained from neonates 24 and 72 hours after birth. Clinical signs such as respiratory distress, milk intolerance, and decreased reflexes were also recorded. In the absence of contraindications to breastfeeding, the infant was fed with her or his breast milk. After 72 hours, if the baby has stabilized clinical condition and requires no hospitalization, she/he would be discharged. An outpatient visit was made to infants in the first week of discharge. The neonate clinical outcomes were followed for four weeks. Furthermore, at the end of the first month, newborns' general condition and weight were requested over the phone. Infants requiring serum and antibiotics for any reason (e.g., prematurity, treatment for respiratory distress, and clinical sepsis) were visited twice in the first month following discharge.

### 2.2. Data Collection

The epidemiological, clinical presentations such as respiratory distress, milk intolerance, decreased reflexes, laboratory and radiological findings, medications, and outcomes data were collected with a data collection checklist from electronic medical records. Two trained nurses reviewed all data.

### 2.3. RT-PCR Assay for SARS-CoV-2 Detection

Nasopharyngeal/throat swabs were collected and analyzed in mothers and neonates for SARS-CoV-2 RNA using RT-PCR. Also, viral RNA was freshly extracted using the Ribospin vRD plus Kit (GeneAll, Seoul, South Korea) according to the manufacturer's instructions. Isolated RNA was analyzed by LightMix® SarbecoV E-gene kit (TIB Molbiol, Berlin, Germany) with LightCycler Multiplex RNA Virus Master (Roche). Specimen collection and laboratory testing followed WHO guidance [11, 12].

### 2.4. Statistical Analysis

Data were analyzed using SPSS version 16. Continuous variables were expressed as the range. Also, categorical variables were expressed as number (%).

## 3. Results

**Figure 1:**
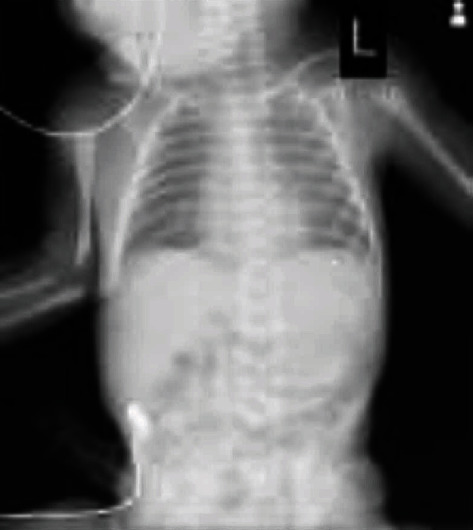
Chest radiograph of preterm infant with respiratory distress syndrome (RDS).

**Figure 2:**
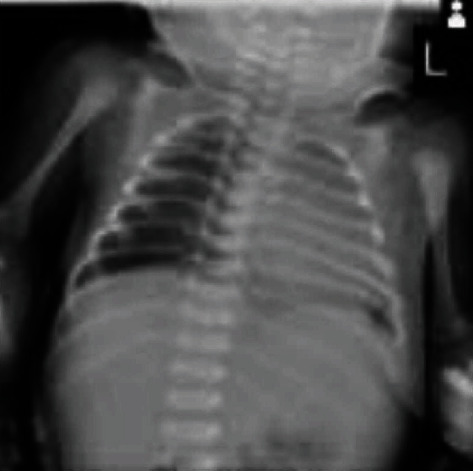
Chest radiograph of the newborn with diagnosis of transient tachypnea of the newborn (TTN).

In this study, eleven neonates born to mothers strongly probable or confirmed COVID-19, three of them were transferred to another center after delivery due to the request of their parents, and finally, eight neonates were identified and included in the current study. The mean gestational age and birth weight of newborns were 37 ± 3.19 weeks (30^₊6^-40) and 3,077.50 ± 697.64 g (1720-3900), respectively (Table 1). None of the newborns required resuscitation in the delivery room, and only one preterm infant was supported by continuous positive airway pressure (CPAP). Apgar scored in the first and fifth minute in all neonates at ≥8 and ≥9 out of 10, respectively. Three infants required antibiotic therapy (ampicillin and amikacin) due to respiratory distress in two cases and vomiting and feeding intolerance in one. One of the two cases with respiratory distress was a preterm infant who also received surfactant (Curosurf). Another case was a neonate with 38 weeks gestational with a diagnosis of transient tachypnea of the newborn (TTN) (Figures 1 and 2). In these three cases, a high concentration of C-reactive protein (CRP) (35, 16, and 48 mg/dL) was reported, but routine blood culture was negative in these three babies. In the follow-up of infants at age four weeks, all babies, except in one case, showed that optimal growth (3,968.75 ± 843.58 g). The infant, who did not weigh properly, was born to a positive PCR and HRCT mother and did not have successful breastfeeding. All babies were breastfed except in two cases. All cases were in an acceptable sensory-motor condition with standard auditory otoacoustic emissions (OAE) at four weeks of age.

It is important to note that 23 mothers were admitted with fever and labor pains, and 11 mothers had a highly probable or confirmed diagnosis of COVID-19. These mothers were singleton pregnancies, and three of them were preterm and others more than 37 weeks of gestation. Six women were tested positive for SARS-CoV-2 PCR, and the remaining five showed suggestive results of pulmonary HRCT. A mother had dyspnea and lymphopenia with pulmonary involvement in HRCT despite negative RT-PCR tests. For this reason, the pregnancy ended 36 weeks into gestational age. Six mothers had CRP levels greater than 15 mg/dL (two cases with more than 100 mg/dL). Only in 3 cases were amniotic fluid samples successfully sent for PCR analysis, and all were negative. Of note, these three cases were only compatible with HRCT for COVID-19.

## 4. Discussion

There are currently reported cases of newborns born to mothers with COVID-19. This study is a retrospective cohort of neonatal outcomes for pregnant women with COVID-19. The current study is the first to report neonatal outcomes of pregnant women infected with COVID-19 in Babol, North of Iran, at the end of the first regional peak of the virus. We have described eight neonates of mothers affected by COVID-19. Like other studies reported to date, our neonates were asymptomatic or had mild to moderate clinical signs [13, 14]. Common clinical presentations of symptomatic neonates were respiratory distress, tachypnea, vomiting, and feeding intolerance. This manifestation and high serum CRP levels in three infants are common in neonatal sepsis. Meanwhile, routine blood cultures were all negative. They have been successfully treated with our standard treatment.

As shown, early antibiotic therapy to prevent secondary bacterial infections may decrease complications and mortality. Zeng et al. reported that neonates born to mothers with COVID-19 were suspected of having sepsis that had improved with antibiotic treatment [15]. These neonates had a pattern of clinical characteristics somewhat similar to other reports, and also, they had favorable outcomes.

The most common symptoms among the mothers participating in the study were fever and dyspnea. Laboratory findings indicated that the level of CRP and lymphopenia had increased. In other words, our pregnant women had clinical characteristics and laboratory results almost similar to those described in nonpregnant women infected with COVID-19 [16]. Unfortunately, we were able to test amniotic fluid samples only in 3 PCR-negative cases.

Literature review for other coronavirus infections in pregnancy showed that the mother's infection led to increased obstetric complications (stillbirth, intrauterine fetal death, and preterm delivery). However, the virus was not isolated from the neonatal sample [17]. Besides, the placenta is a source of the virus in the Ebola virus infection, making vertical transmission possible [18]. Also, in another case report, the mother had severe acute respiratory syndrome (SARS) during pregnancy, and there was an antibody in the serum. However, cord blood, placental samples, breast milk, and infant stool samples were all negative for viruses and antibodies [19].

Our findings consisted of some studies that have presented no notable clinical symptoms suggestive of COVID-19 infection in these neonates, and also, samples, such as amniotic fluid, were negative for SARS-CoV-2 [13, 20]. However, with these findings, we cannot rule out or prove the possibility of vertical transmission of the infection, requiring further research. To the best of our knowledge, by August 13, 2020, there have been 16,798 confirmed cases of pregnant women in the U.S. with COVID-19, including 37 deaths, reported to the centers for disease control and prevention (CDC).

In our study, there were no maternal and neonatal deaths due to COVID-19 infection. It could be associated with quick delivery or small sample size. The limitations of this study were the small sample size and some limitations of the clinical data. Furthermore, due to the relatively short time since the occurrence of this pandemic, the long-term outcomes of the neonates need to be further investigated.

## 5. Conclusions

Based on the recent study, there is no evidence of intrauterine or peripartum transmission of COVID-19 from mothers to their children.

## Figures and Tables

**Table 1:** Baseline characteristics of 8 newborns with mothers with COVID-19.

	Neonate1	Neonate2	Neonate3	Neonate4	Neonate5	Neonate6	Neonate7	Neonate8
Gestational age at delivery	40 weeks	34 weeks	36^+1^ weeks	40 weeks	39 weeks	38 weeks	38 weeks	30^+6^ weeks
Birth weight (grams)	3830	2850	2660	3340	39	3240	3080	1720
Root of delivery	C/S	C/S	C/S	C/S	NVD	C/S	NVD	C/S
Apgar score	10/10	9/10	9/10	9/10	9/10	8/10	9/10	8/9
Need for resuscitation at birth	No	No	No	No	No	No	No	Only needs CPAP
Respiratory distress	No	No	No	No	No	No	Yes	Yes
Feeding problem	No	No	No	No	No	Yes	No	Yes
Poor reflexes	No	No	No	No	No	Yes	Yes	No
Weight at age 4 weeks	4200	3670	4850	4150	3900	4650	4230	2100
Nutrition during 4 weeks	BMF	Formula	BMF	BMF	BMF+formula	BMF	BMF	BMF+formula

Abbreviations: C/S: cesarean section; NVD: normal vaginal delivery; CPAP: continuous positive airway pressure; BMF: breast milk fortifier.

## Data Availability

The data will be available on request through contact to request data from the corresponding author.
